# Jérôme Lejeune passed away 25 years ago

**DOI:** 10.1186/s41065-019-0094-8

**Published:** 2019-06-06

**Authors:** Vicente Soriano

**Affiliations:** UNIR Health Sciences School & Medical Center, calle Almansa 101, 28040 Madrid, Spain

**Keywords:** Trisomy 21, Jérôme Lejeune, Genetic diagnosis, Gene editing

## Background

During the past 60 years, modern genetics has steadily evolved from diagnostics to therapeutics. However, treatment of genetic disorders is still in its infancy, with the advent of genome editing as its greatest promise.

Genetic diagnosis was formally initiated in 1959 by Jérôme Lejeune (Paris, 1926–1994), who reported for the first time that trisomy 21 was the cause of the Down syndrome, the most frequent aneuploid anomaly in human newborns, characterized by intellectual disability and physical malformations [1]. After this pivotal discovery, many other chromosomopathies -such as the “cri du chat” syndrome- and distinct genetic abnormalities that produce congenital human illnesses were described by Lejeune’s team [2, 3].

Initially using karyotyping as the method for identifying visible chromosomal anomalies, the technique was only ready for chromosomal aberrations observable by the naked eye or via the microscope. Although genetic diagnosis does not cure a disease, it constitutes a tremendous step forward in the way patients could be subsequently treated. Furthermore, understanding and providing a tag that describes a particular genetic disease precludes from undergoing unnecessary treatments, and it is also a source of help for the patient and families via charitable organizations or governmental health support.

## Main text

Over time, genetic diagnosis has largely replaced karyotyping by genotyping, a switch that was largely facilitated after a second major breakthrough occurred in 2001, when the complete sequencing of the human genome was achieved, encompassing roughly 3200 million base pairs [4, 5]. Intriguingly, only 1.5% -about 20,000 genes-, correspond to coding regions, a proportion much lower than originally expected. Nowadays we are learning how non-coding regions plays an important role in the regulation and expression of gene functions, as evidenced by ongoing projects such as ENCODE (www.encodeproject.org).

Arguably, the latest major advancement in human genetics came in 2012, following the discovery of the CRISPR/Cas9 system as a mechanism for adaptive immunity in bacteria and archaea, and the recognition of its potential for gene editing, meaning insertion, deletion or replacement of small DNA fragments [6]. The door is now open for modifying human DNA in an unprecedented fashion, including efforts to treat genetic disorders.

**Fig. 1:**
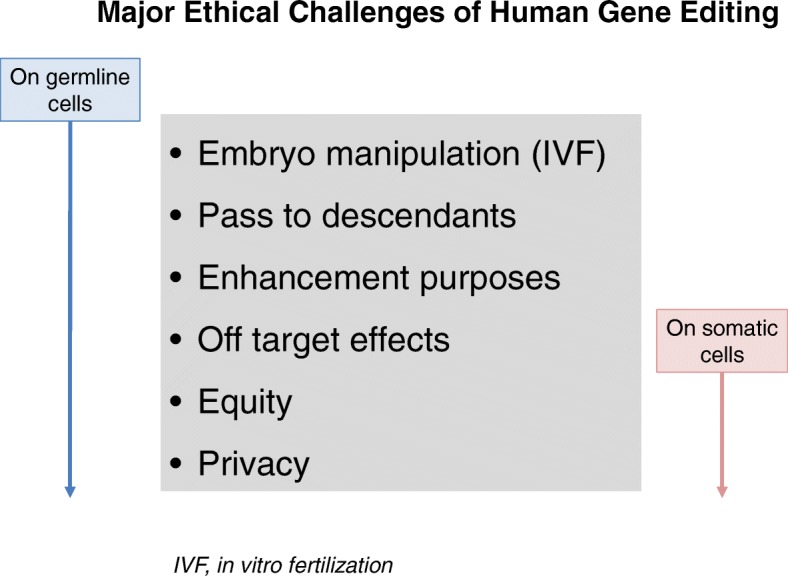
Major Ethical Challenges of Human Gene Editing

The application of gene editing to human somatic cells is especially being tested for monogenic conditions that have recessive inheritance. More complexities exist for using the CRISPR/Cas9 technology for correcting polygenic diseases. Major caveats are off-target effects, as result of non-specific events on other parts of the genome; low-target effects, when just a proportion of the DNA sequence matches are corrected; and unknown target effects, when gene interactions that were not predicted may occur. In addition, questions about equity (equal access for all) and privacy must be considered (Fig. 1).

Theoretically, gene editing may be performed at various developmental stages of the human being. First, it can be delivered into one-cell embryos or oocytes at the time of intracytoplasmic sperm injection, in an attempt to minimize mosaicism. Second, it may be delivered into embryos before completing organogenesis during the first trimester of pregnancy. Third, it can be done on maturing fetuses before birth. Fourth, in vivo gene editing can be performed in newborns, children or adults using the appropriate vectors to correct or alleviate clinical manifestations. In the latter case, clinical trials using somatic gene editing have already started in Europe and the United States as effective treatment and cure for some monogenic conditions and cancers. CRISPR/Cas9 technology is also being tested for eliminating viral infections that establish chronic infection of human cells, such as HIV or hepatitis B.

Gene editing in utero might allow to correct de novo mutations, which are generated in embryos/fetuses, rather than those inherited from parents before birth. Almost one third of all genetic diseases are caused by de novo mutations. Using non-invasive prenatal diagnosis, de novo mutations can now be detected in the cell-free fetal DNA that circulates in the maternal blood. In utero gene editing will allow for correction before birth and might be ethically acceptable whereas editing human embryos obtained using in vitro fertilization will not. Furthermore, edited genes in fetuses will not be germ-line transmissible unless germ cells are targeted.

Given that Jérôme Lejeune, considered the father of modern genetics, passed away 25 years ago and 2019 marks the 60th anniversary of his seminal contribution to genetic science, new hopes for congenital illnesses using gene editing can be viewed as an extension of Lejeune’s research on the genetic basis for the Down syndrome. At this pivotal moment in scientific development where Dr. He Jiankui, a Chinese scientist, recently reported that two human twin girls had been born following in vitro fertilization of gene editing embryos from a progenitor male that was HIV-infected, the medical community has followed an unprecedented response, unanimously proclaiming that such kind of experiments should never be done again. The absence of justification and regulation of human gene editing, specially involving germline cells, thus could have devastating consequences for the human species [7, 8].

Lejeune tried during his life to find a cure rather than supporting prenatal diagnosis for eugenic abortion [9]. As part of his legacy, the Lejeune’s Foundation continues its commitment for providing care to persons with genetic intellectual disability syndromes and their families around the world (www.fondationlejeune.org/). The pioneer clinic in Paris is a notable example, which will be soon reproduced by clinics in Washington DC and Madrid. Finally, new promising results for some genetic illnesses using the CRISPR/Cas9 technology hopefully will sometime open the opportunity for treating trisomy 21 before birth.

## Conclusions

Lejeune’s reflection was always on the side of the patient, especially the unborn person [9]. His famous lecture “On the nature of men”, delivered when he received the William Allen Award, granted by the American Society of Human Genetics [10], is an impressive testimony about how ethics must guide human research. For his work, the US president J.F. Kennedy personally honored him with the Kennedy Prize. Given that 2019 marks the 25th anniversary of Lejeune’s passing away, his words should inspire discussions arisen about human germline editing.

## Data Availability

Yes, at request.

## Abbreviations

Cas9: CRISPR-associated protein 9
CRISPR: Clustered regularly interspaced short palindromic repeat
